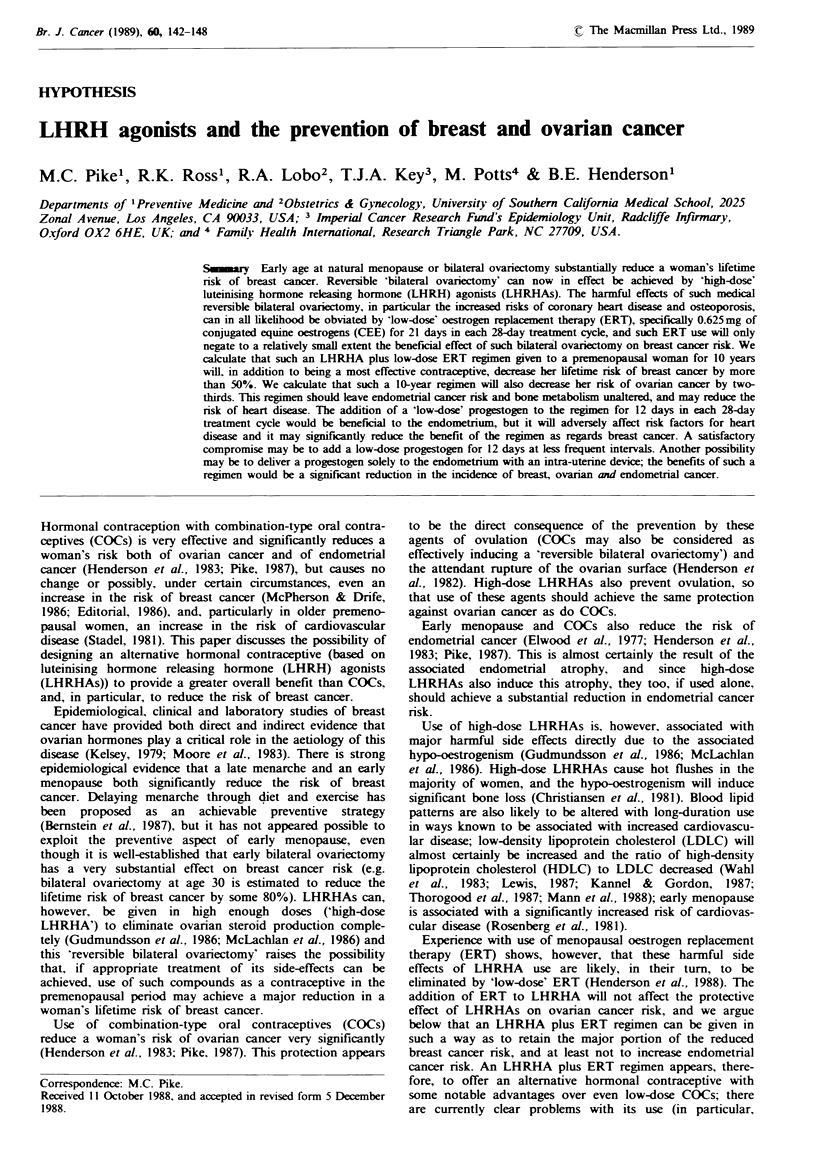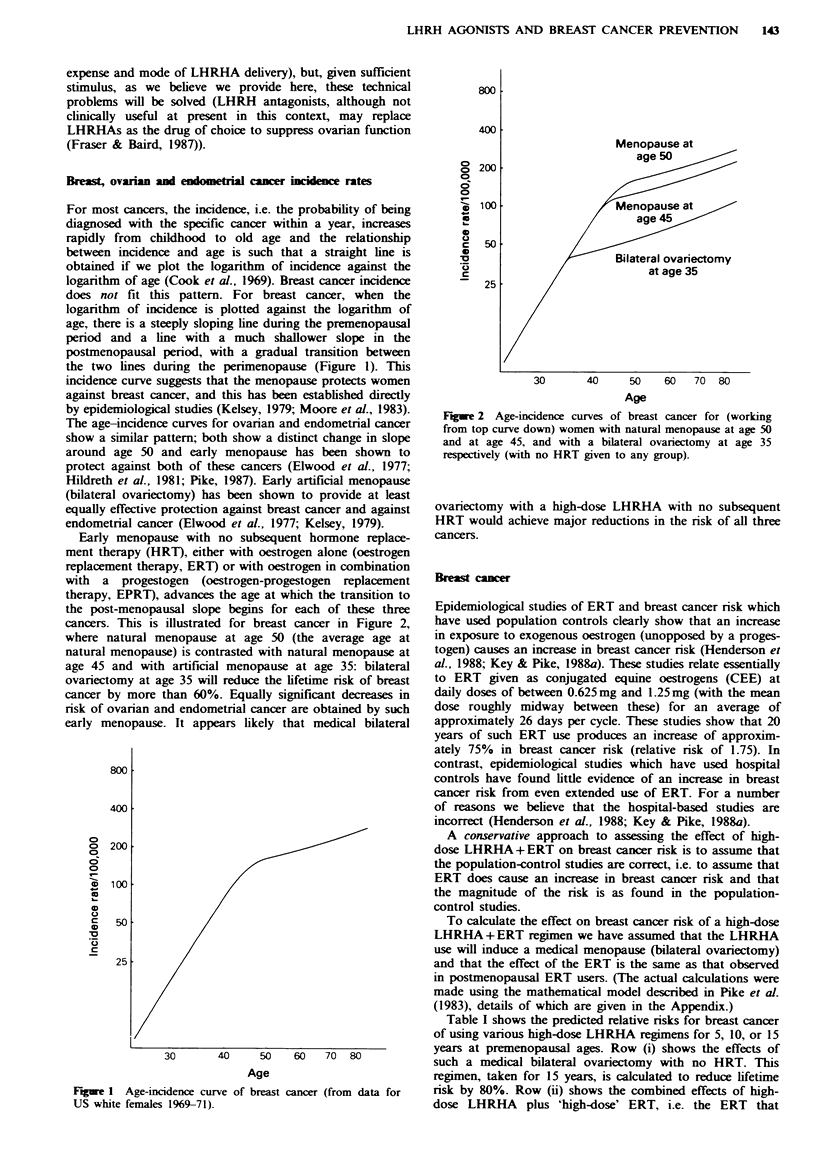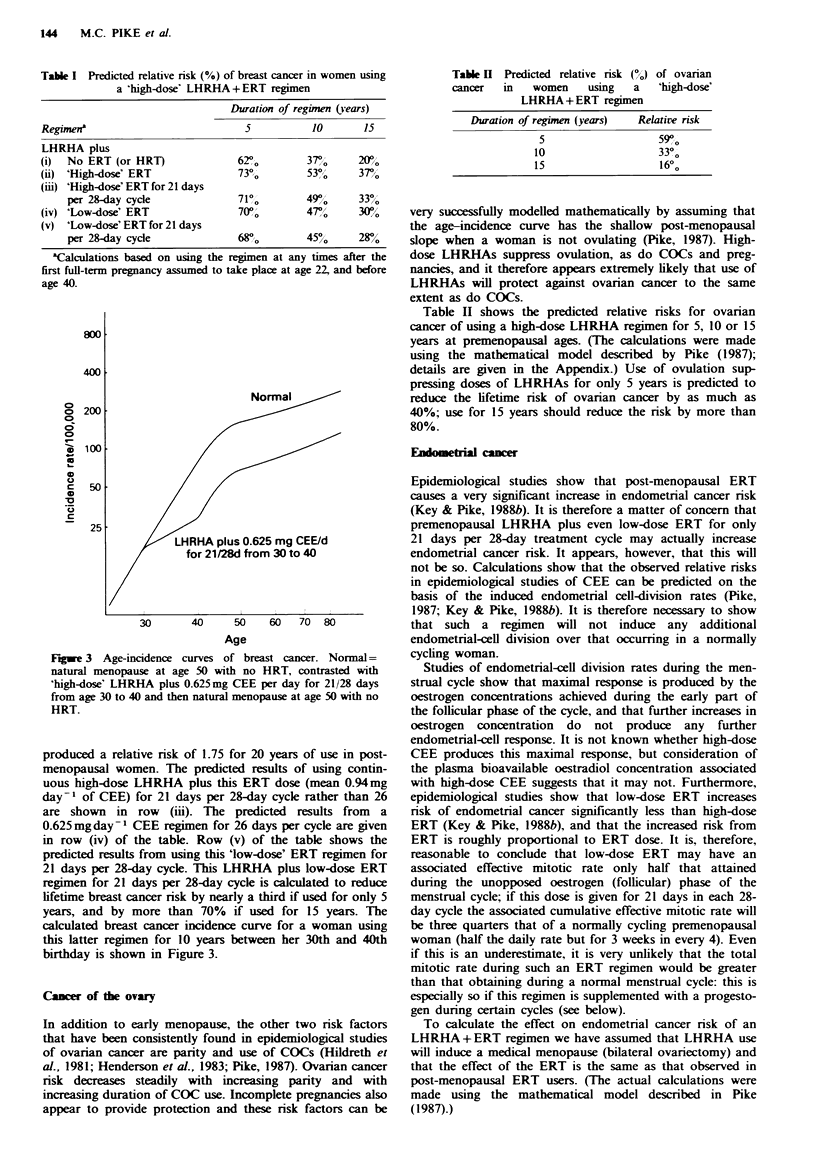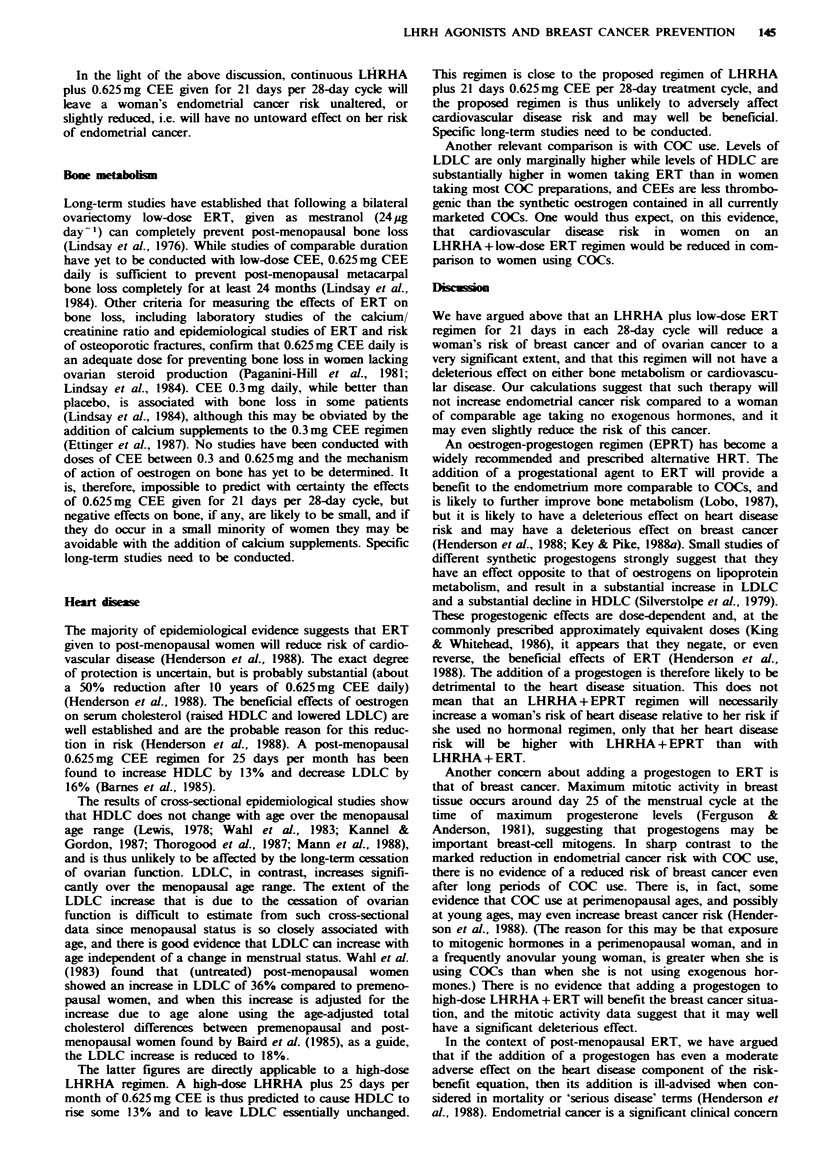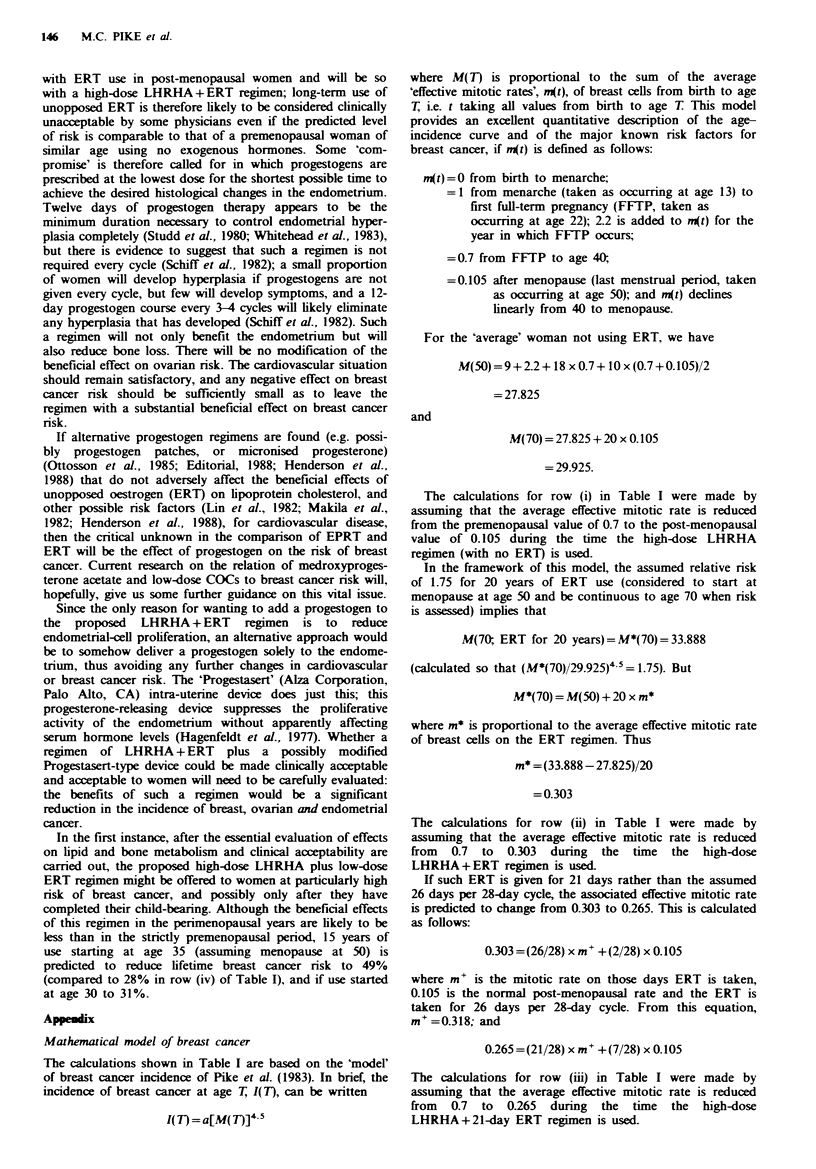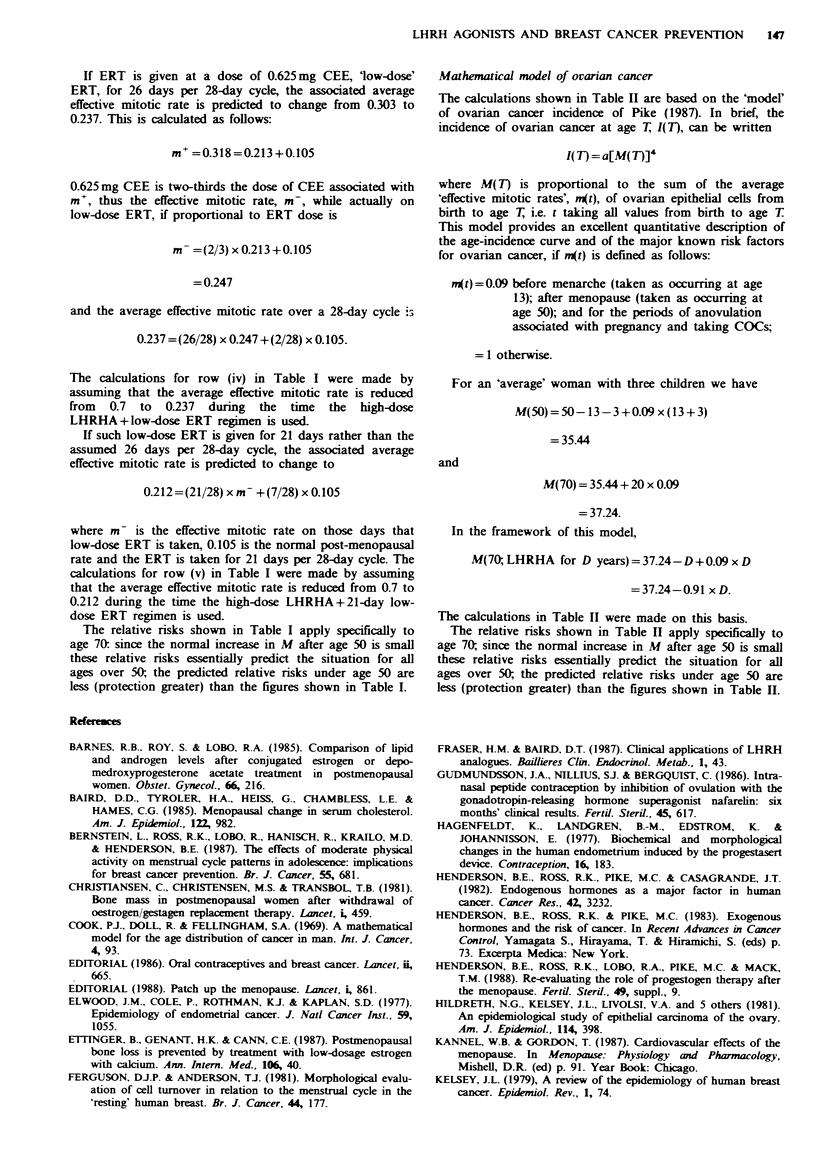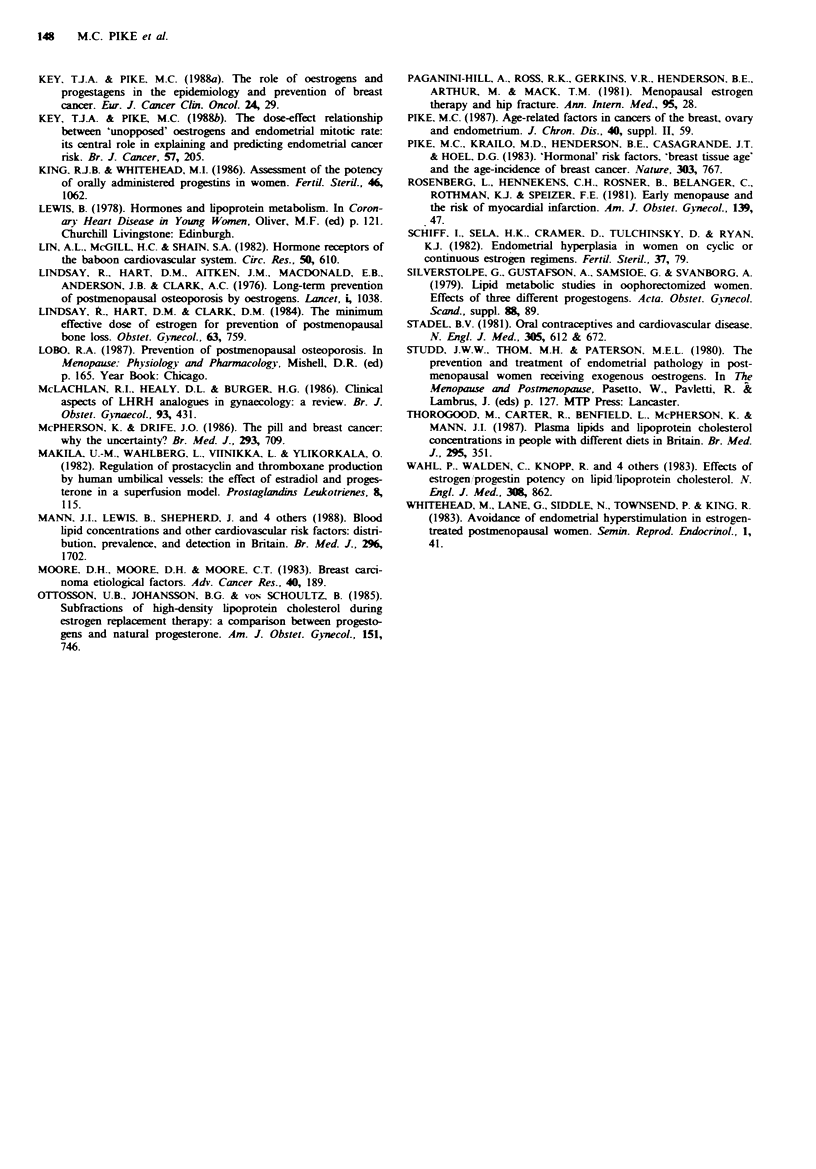# LHRH agonists and the prevention of breast and ovarian cancer.

**DOI:** 10.1038/bjc.1989.237

**Published:** 1989-07

**Authors:** M. C. Pike, R. K. Ross, R. A. Lobo, T. J. Key, M. Potts, B. E. Henderson

**Affiliations:** Department of Preventive Medicine, University of Southern California Medical School, Los Angeles 90033.

## Abstract

Early age at natural menopause or bilateral ovariectomy substantially reduce a woman's lifetime risk of breast cancer. Reversible 'bilateral ovariectomy' can now in effect be achieved by 'high-dose' luteinising hormone releasing hormone (LHRH) agonists (LHRHAs). The harmful effects of such medical reversible bilateral ovariectomy, in particular the increased risks of coronary heart disease and osteoporosis, can in all likelihood be obviated by 'low-dose' oestrogen replacement therapy (ERT), specifically 0.625 mg of conjugated equine oestrogens (CEE) for 21 days in each 28-day treatment cycle, and such ERT use will only negate to a relatively small extent the beneficial effect of such bilateral ovariectomy on breast cancer risk. We calculate that such an LHRHA plus low-dose ERT regimen given to a premenopausal woman for 10 years will, in addition to being a most effective contraceptive, decrease her lifetime risk of breast cancer by more than 50%. We calculate that such a 10-year regimen will also decrease her risk of ovarian cancer by two-thirds. This regimen should leave endometrial cancer risk and bone metabolism unaltered, and may reduce the risk of heart disease. The addition of a 'low-dose' progestogen to the regimen for 12 days in each 28-day treatment cycle would be beneficial to the endometrium, but it will adversely affect risk factors for heart disease and it may significantly reduce the benefit of the regimen as regards breast cancer. A satisfactory compromise may be to add a low-dose progestogen for 12 days at less frequent intervals. Another possibility may be to deliver a progestogen solely to the endometrium with an intra-uterine device; the benefits of such a regimen would be a significant reduction in the incidence of breast, ovarian and endometrial cancer.


Br. J. Cancer (1989), 60, 142-148 © The Macmillan Press Ltd., 1989

HYPOTHESIS

### LHRH agonists and the prevention of breast and ovarian cancer

M.C. Pike[1], R.K. Ross[1], R.A. Lobo[2], T.J.A. Key[3], M. Potts[4] & B.E. Henderson[1]

*Departments of [1]Preventive Medicine and [2]Obstetrics & Gynecology, University of Southern California Medical School, 2025 Zonal Avenue, Los Angeles, CA 90033, USA; [3] Imperial Cancer Research Fund's Epidemiology Unit, Radcliffe Infirmary, Oxford OX2 6HE, UK; and [4] Family Health International, Research Triangle Park, NC 27709, USA.*

**Summary** Early age at natural menopause or bilateral ovariectomy substantially reduce a woman's lifetime risk of breast cancer. Reversible 'bilateral ovariectomy' can now in effect be achieved by 'high-dose' luteinising hormone releasing hormone (LHRH) agonists (LHRHAs). The harmful effects of such medical reversible bilateral ovariectomy, in particular the increased risks of coronary heart disease and osteoporosis, can in all likelihood be obviated by 'low-dose' oestrogen replacement therapy (ERT), specifically 0.625 mg of conjugated equine oestrogens (CEE) for 21 days in each 28-day treatment cycle, and such ERT use will only negate to a relatively small extent the beneficial effect of such bilateral ovariectomy on breast cancer risk. We calculate that such an LHRHA plus low-dose ERT regimen given to a premenopausal woman for 10 years will, in addition to being a most effective contraceptive, decrease her lifetime risk of breast cancer by more than 50%. We calculate that such a 10-year regimen will also decrease her risk of ovarian cancer by two-thirds. This regimen should leave endometrial cancer risk and bone metabolism unaltered, and may reduce the risk of heart disease. The addition of a 'low-dose' progestogen to the regimen for 12 days in each 28-day treatment cycle would be beneficial to the endometrium, but it will adversely affect risk factors for heart disease and it may significantly reduce the benefit of the regimen as regards breast cancer. A satisfactory compromise may be to add a low-dose progestogen for 12 days at less frequent intervals. Another possibility may be to deliver a progestogen solely to the endometrium with an intra-uterine device; the benefits of such a regimen would be a significant reduction in the incidence of breast, ovarian *and* endometrial cancer.

Hormonal contraception with combination-type oral contraceptives (COCs) is very effective and significantly reduces a woman's risk both of ovarian cancer and of endometrial cancer (Henderson *et al.*, 1983; Pike, 1987), but causes no change or possibly, under certain circumstances, even an increase in the risk of breast cancer (McPherson & Drife, 1986; Editorial, 1986), and, particularly in older premenopausal women, an increase in the risk of cardiovascular disease (Stadel, 1981). This paper discusses the possibility of designing an alternative hormonal contraceptive (based on luteinising hormone releasing hormone (LHRH) agonists (LHRHAs)) to provide a greater overall benefit than COCs, and, in particular, to reduce the risk of breast cancer.

Epidemiological, clinical and laboratory studies of breast cancer have provided both direct and indirect evidence that ovarian hormones play a critical role in the aetiology of this disease (Kelsey, 1979; Moore *et al.*, 1983). There is strong epidemiological evidence that a late menarche and an early menopause both significantly reduce the risk of breast cancer. Delaying menarche through diet and exercise has been proposed as an achievable preventive strategy (Bernstein *et al.*, 1987), but it has not appeared possible to exploit the preventive aspect of early menopause, even though it is well-established that early bilateral ovariectomy has a very substantial effect on breast cancer risk (e.g. bilateral ovariectomy at age 30 is estimated to reduce the lifetime risk of breast cancer by some 80%). LHRHAs can, however, be given in high enough doses ('high-dose LHRHA') to eliminate ovarian steroid production completely (Gudmundsson *et al.*, 1986; McLachlan *et al.*, 1986) and this 'reversible bilateral ovariectomy' raises the possibility that, if appropriate treatment of its side-effects can be achieved, use of such compounds as a contraceptive in the premenopausal period may achieve a major reduction in a woman's lifetime risk of breast cancer.

Use of combination-type oral contraceptives (COCs) reduce a woman's risk of ovarian cancer very significantly (Henderson *et al.*, 1983; Pike, 1987). This protection appears to be the direct consequence of the prevention by these agents of ovulation (COCs may also be considered as effectively inducing a 'reversible bilateral ovariectomy') and the attendant rupture of the ovarian surface (Henderson *et al.*, 1982). High-dose LHRHAs also prevent ovulation, so that use of these agents should achieve the same protection against ovarian cancer as do COCs.

Early menopause and COCs also reduce the risk of endometrial cancer (Elwood *et al.*, 1977; Henderson *et al.*, 1983; Pike, 1987). This is almost certainly the result of the associated endometrial atrophy, and since high-dose LHRHAs also induce this atrophy, they too, if used alone, should achieve a substantial reduction in endometrial cancer risk.

Use of high-dose LHRHAs is, however, associated with major harmful side effects directly due to the associated hypo-oestrogenism (Gudmundsson *et al.*, 1986; McLachlan *et al.*, 1986). High-dose LHRHAs cause hot flushes in the majority of women, and the hypo-oestrogenism will induce significant bone loss (Christiansen *et al.*, 1981). Blood lipid patterns are also likely to be altered with long-duration use in ways known to be associated with increased cardiovascular disease; low-density lipoprotein cholesterol (LDLC) will almost certainly be increased and the ratio of high-density lipoprotein cholesterol (HDLC) to LDLC decreased (Wahl *et al.*, 1983; Lewis, 1987; Kannel & Gordon, 1987; Thorogood *et al.*, 1987; Mann *et al.*, 1988); early menopause is associated with a significantly increased risk of cardiovascular disease (Rosenberg *et al.*, 1981).

Experience with use of menopausal oestrogen replacement therapy (ERT) shows, however, that these harmful side effects of LHRHA use are likely, in their turn, to be eliminated by 'low-dose' ERT (Henderson *et al.*, 1988). The addition of ERT to LHRHA will not affect the protective effect of LHRHAs on ovarian cancer risk, and we argue below that an LHRHA plus ERT regimen can be given in such a way as to retain the major portion of the reduced breast cancer risk, and at least not to increase endometrial cancer risk. An LHRHA plus ERT regimen appears, therefore, to offer an alternative hormonal contraceptive with some notable advantages over even low-dose COCs; there are currently clear problems with its use (in particular,

Correspondence: M.C. Pike.
Received 11 October 1988, and accepted in revised form 5 December 1988.

expense and mode of LHRHA delivery), but, given sufficient stimulus, as we believe we provide here, these technical problems will be solved (LHRH antagonists, although not clinically useful at present in this context, may replace LHRHAs as the drug of choice to suppress ovarian function (Fraser & Baird, 1987)).

**Breast, ovarian and endometrial cancer incidence rates**

For most cancers, the incidence, i.e. the probability of being diagnosed with the specific cancer within a year, increases rapidly from childhood to old age and the relationship between incidence and age is such that a straight line is obtained if we plot the logarithm of incidence against the logarithm of age (Cook *et al.*, 1969). Breast cancer incidence does *not* fit this pattern. For breast cancer, when the logarithm of incidence is plotted against the logarithm of age, there is a steeply sloping line during the premenopausal period and a line with a much shallower slope in the postmenopausal period, with a gradual transition between the two lines during the perimenopause (Figure 1). This incidence curve suggests that the menopause protects women against breast cancer, and this has been established directly by epidemiological studies (Kelsey, 1979; Moore *et al.*, 1983). The age–incidence curves for ovarian and endometrial cancer show a similar pattern; both show a distinct change in slope around age 50 and early menopause has been shown to protect against both of these cancers (Elwood *et al.*, 1977; Hildreth *et al.*, 1981; Pike, 1987). Early artificial menopause (bilateral ovariectomy) has been shown to provide at least equally effective protection against breast cancer and against endometrial cancer (Elwood *et al.*, 1977; Kelsey, 1979).

Early menopause with no subsequent hormone replacement therapy (HRT), either with oestrogen alone (oestrogen replacement therapy, ERT) or with oestrogen in combination with a progestogen (oestrogen-progestogen replacement therapy, EPRT), advances the age at which the transition to the post-menopausal slope begins for each of these three cancers. This is illustrated for breast cancer in Figure 2, where natural menopause at age 50 (the average age at natural menopause) is contrasted with natural menopause at age 45 and with artificial menopause at age 35: bilateral ovariectomy at age 35 will reduce the lifetime risk of breast cancer by more than 60%. Equally significant decreases in risk of ovarian and endometrial cancer are obtained by such early menopause. It appears likely that medical bilateral ovariectomy with a high-dose LHRHA with no subsequent HRT would achieve major reductions in the risk of all three cancers.

**Figure 1** Age-incidence curve of breast cancer (from data for US white females 1969–71).

**Figure 2** Age-incidence curves of breast cancer for (working from top curve down) women with natural menopause at age 50 and at age 45, and with a bilateral ovariectomy at age 35 respectively (with no HRT given to any group).

**Breast cancer**

Epidemiological studies of ERT and breast cancer risk which have used population controls clearly show that an increase in exposure to exogenous oestrogen (unopposed by a progestogen) causes an increase in breast cancer risk (Henderson *et al.*, 1988; Key & Pike, 1988*a*). These studies relate essentially to ERT given as conjugated equine oestrogens (CEE) at daily doses of between 0.625 mg and 1.25 mg (with the mean dose roughly midway between these) for an average of approximately 26 days per cycle. These studies show that 20 years of such ERT use produces an increase of approximately 75% in breast cancer risk (relative risk of 1.75). In contrast, epidemiological studies which have used hospital controls have found little evidence of an increase in breast cancer risk from even extended use of ERT. For a number of reasons we believe that the hospital-based studies are incorrect (Henderson *et al.*, 1988; Key & Pike, 1988*a*).

A *conservative* approach to assessing the effect of high-dose LHRHA + ERT on breast cancer risk is to assume that the population-control studies are correct, i.e. to assume that ERT does cause an increase in breast cancer risk and that the magnitude of the risk is as found in the population-control studies.

To calculate the effect on breast cancer risk of a high-dose LHRHA + ERT regimen we have assumed that the LHRHA use will induce a medical menopause (bilateral ovariectomy) and that the effect of the ERT is the same as that observed in postmenopausal ERT users. (The actual calculations were made using the mathematical model described in Pike *et al.* (1983), details of which are given in the Appendix.)

Table I shows the predicted relative risks for breast cancer of using various high-dose LHRHA regimens for 5, 10, or 15 years at premenopausal ages. Row (i) shows the effects of such a medical bilateral ovariectomy with no HRT. This regimen, taken for 15 years, is calculated to reduce lifetime risk by 80%. Row (ii) shows the combined effects of high-dose LHRHA plus 'high-dose' ERT, i.e. the ERT that

**Table I** Predicted relative risk (%) of breast cancer in women using a 'high-dose' LHRHA + ERT regimen

| Regimen[a] | Duration of regimen (years) 5 | 10 | 15 |
|---|---|---|---|
| LHRHA plus | | | |
| (i) No ERT (or HRT) | 62% | 37% | 20% |
| (ii) 'High-dose' ERT | 73% | 53% | 37% |
| (iii) 'High-dose' ERT for 21 days per 28-day cycle | 71% | 49% | 33% |
| (iv) 'Low-dose' ERT | 70% | 47% | 30% |
| (v) 'Low-dose' ERT for 21 days per 28-day cycle | 68% | 45% | 28% |

[a]Calculations based on using the regimen at any times after the first full-term pregnancy assumed to take place at age 22, and before age 40.

**Figure 3** Age-incidence curves of breast cancer. Normal = natural menopause at age 50 with no HRT, contrasted with 'high-dose' LHRHA plus 0.625 mg CEE per day for 21/28 days from age 30 to 40 and then natural menopause at age 50 with no HRT.

produced a relative risk of 1.75 for 20 years of use in post-menopausal women. The predicted results of using continuous high-dose LHRHA plus this ERT dose (mean 0.94 mg day$^{-1}$ of CEE) for 21 days per 28-day cycle rather than 26 are shown in row (iii). The predicted results from a 0.625 mg day$^{-1}$ CEE regimen for 26 days per cycle are given in row (iv) of the table. Row (v) of the table shows the predicted results from using this 'low-dose' ERT regimen for 21 days per 28-day cycle. This LHRHA plus low-dose ERT regimen for 21 days per 28-day cycle is calculated to reduce lifetime breast cancer risk by nearly a third if used for only 5 years, and by more than 70% if used for 15 years. The calculated breast cancer incidence curve for a woman using this latter regimen for 10 years between her 30th and 40th birthday is shown in Figure 3.

### Cancer of the ovary

In addition to early menopause, the other two risk factors that have been consistently found in epidemiological studies of ovarian cancer are parity and use of COCs (Hildreth et al., 1981; Henderson et al., 1983; Pike, 1987). Ovarian cancer risk decreases steadily with increasing parity and with increasing duration of COC use. Incomplete pregnancies also appear to provide protection and these risk factors can be very successfully modelled mathematically by assuming that the age–incidence curve has the shallow post-menopausal slope when a woman is not ovulating (Pike, 1987). High-dose LHRHAs suppress ovulation, as do COCs and pregnancies, and it therefore appears extremely likely that use of LHRHAs will protect against ovarian cancer to the same extent as do COCs.

**Table II** Predicted relative risk (%) of ovarian cancer in women using a 'high-dose' LHRHA + ERT regimen

| Duration of regimen (years) | Relative risk |
|---|---|
| 5 | 59% |
| 10 | 33% |
| 15 | 16% |

Table II shows the predicted relative risks for ovarian cancer of using a high-dose LHRHA regimen for 5, 10 or 15 years at premenopausal ages. (The calculations were made using the mathematical model described by Pike (1987); details are given in the Appendix.) Use of ovulation suppressing doses of LHRHAs for only 5 years is predicted to reduce the lifetime risk of ovarian cancer by as much as 40%; use for 15 years should reduce the risk by more than 80%.

### Endometrial cancer

Epidemiological studies show that post-menopausal ERT causes a very significant increase in endometrial cancer risk (Key & Pike, 1988b). It is therefore a matter of concern that premenopausal LHRHA plus even low-dose ERT for only 21 days per 28-day treatment cycle may actually increase endometrial cancer risk. It appears, however, that this will not be so. Calculations show that the observed relative risks in epidemiological studies of CEE can be predicted on the basis of the induced endometrial cell-division rates (Pike, 1987; Key & Pike, 1988b). It is therefore necessary to show that such a regimen will not induce any additional endometrial-cell division over that occurring in a normally cycling woman.

Studies of endometrial-cell division rates during the menstrual cycle show that maximal response is produced by the oestrogen concentrations achieved during the early part of the follicular phase of the cycle, and that further increases in oestrogen concentration do not produce any further endometrial-cell response. It is not known whether high-dose CEE produces this maximal response, but consideration of the plasma bioavailable oestradiol concentration associated with high-dose CEE suggests that it may not. Furthermore, epidemiological studies show that low-dose ERT increases risk of endometrial cancer significantly less than high-dose ERT (Key & Pike, 1988b), and that the increased risk from ERT is roughly proportional to ERT dose. It is, therefore, reasonable to conclude that low-dose ERT may have an associated effective mitotic rate only half that attained during the unopposed oestrogen (follicular) phase of the menstrual cycle; if this dose is given for 21 days in each 28-day cycle the associated cumulative effective mitotic rate will be three quarters that of a normally cycling premenopausal woman (half the daily rate but for 3 weeks in every 4). Even if this is an underestimate, it is very unlikely that the total mitotic rate during such an ERT regimen would be greater than that obtaining during a normal menstrual cycle: this is especially so if this regimen is supplemented with a progestogen during certain cycles (see below).

To calculate the effect on endometrial cancer risk of an LHRHA + ERT regimen we have assumed that LHRHA use will induce a medical menopause (bilateral ovariectomy) and that the effect of the ERT is the same as that observed in post-menopausal ERT users. (The actual calculations were made using the mathematical model described in Pike (1987).)

In the light of the above discussion, continuous LHRHA plus 0.625 mg CEE given for 21 days per 28-day cycle will leave a woman's endometrial cancer risk unaltered, or slightly reduced, i.e. will have no untoward effect on her risk of endometrial cancer.

### Bone metabolism

Long-term studies have established that following a bilateral ovariectomy low-dose ERT, given as mestranol (24 $\mu$g day$^{-1}$) can completely prevent post-menopausal bone loss (Lindsay *et al.*, 1976). While studies of comparable duration have yet to be conducted with low-dose CEE, 0.625 mg CEE daily is sufficient to prevent post-menopausal metacarpal bone loss completely for at least 24 months (Lindsay *et al.*, 1984). Other criteria for measuring the effects of ERT on bone loss, including laboratory studies of the calcium/creatinine ratio and epidemiological studies of ERT and risk of osteoporotic fractures, confirm that 0.625 mg CEE daily is an adequate dose for preventing bone loss in women lacking ovarian steroid production (Paganini-Hill *et al.*, 1981; Lindsay *et al.*, 1984). CEE 0.3 mg daily, while better than placebo, is associated with bone loss in some patients (Lindsay *et al.*, 1984), although this may be obviated by the addition of calcium supplements to the 0.3 mg CEE regimen (Ettinger *et al.*, 1987). No studies have been conducted with doses of CEE between 0.3 and 0.625 mg and the mechanism of action of oestrogen on bone has yet to be determined. It is, therefore, impossible to predict with certainty the effects of 0.625 mg CEE given for 21 days per 28-day cycle, but negative effects on bone, if any, are likely to be small, and if they do occur in a small minority of women they may be avoidable with the addition of calcium supplements. Specific long-term studies need to be conducted.

### Heart disease

The majority of epidemiological evidence suggests that ERT given to post-menopausal women will reduce risk of cardiovascular disease (Henderson *et al.*, 1988). The exact degree of protection is uncertain, but is probably substantial (about a 50% reduction after 10 years of 0.625 mg CEE daily) (Henderson *et al.*, 1988). The beneficial effects of oestrogen on serum cholesterol (raised HDLC and lowered LDLC) are well established and are the probable reason for this reduction in risk (Henderson *et al.*, 1988). A post-menopausal 0.625 mg CEE regimen for 25 days per month has been found to increase HDLC by 13% and decrease LDLC by 16% (Barnes *et al.*, 1985).

The results of cross-sectional epidemiological studies show that HDLC does not change with age over the menopausal age range (Lewis, 1978; Wahl *et al.*, 1983; Kannel & Gordon, 1987; Thorogood *et al.*, 1987; Mann *et al.*, 1988), and is thus unlikely to be affected by the long-term cessation of ovarian function. LDLC, in contrast, increases significantly over the menopausal age range. The extent of the LDLC increase that is due to the cessation of ovarian function is difficult to estimate from such cross-sectional data since menopausal status is so closely associated with age, and there is good evidence that LDLC can increase with age independent of a change in menstrual status. Wahl *et al.* (1983) found that (untreated) post-menopausal women showed an increase in LDLC of 36% compared to premenopausal women, and when this increase is adjusted for the increase due to age alone using the age-adjusted total cholesterol differences between premenopausal and post-menopausal women found by Baird *et al.* (1985), as a guide, the LDLC increase is reduced to 18%.

The latter figures are directly applicable to a high-dose LHRHA regimen. A high-dose LHRHA plus 25 days per month of 0.625 mg CEE is thus predicted to cause HDLC to rise some 13% and to leave LDLC essentially unchanged. This regimen is close to the proposed regimen of LHRHA plus 21 days 0.625 mg CEE per 28-day treatment cycle, and the proposed regimen is thus unlikely to adversely affect cardiovascular disease risk and may well be beneficial. Specific long-term studies need to be conducted.

Another relevant comparison is with COC use. Levels of LDLC are only marginally higher while levels of HDLC are substantially higher in women taking ERT than in women taking most COC preparations, and CEEs are less thrombogenic than the synthetic oestrogen contained in all currently marketed COCs. One would thus expect, on this evidence, that cardiovascular disease risk in women on an LHRHA + low-dose ERT regimen would be reduced in comparison to women using COCs.

### Discussion

We have argued above that an LHRHA plus low-dose ERT regimen for 21 days in each 28-day cycle will reduce a woman's risk of breast cancer and of ovarian cancer to a very significant extent, and that this regimen will not have a deleterious effect on either bone metabolism or cardiovascular disease. Our calculations suggest that such therapy will not increase endometrial cancer risk compared to a woman of comparable age taking no exogenous hormones, and it may even slightly reduce the risk of this cancer.

An oestrogen-progestogen regimen (EPRT) has become a widely recommended and prescribed alternative HRT. The addition of a progestational agent to ERT will provide a benefit to the endometrium more comparable to COCs, and is likely to further improve bone metabolism (Lobo, 1987), but it is likely to have a deleterious effect on heart disease risk and may have a deleterious effect on breast cancer (Henderson *et al.*, 1988; Key & Pike, 1988*a*). Small studies of different synthetic progestogens strongly suggest that they have an effect opposite to that of oestrogens on lipoprotein metabolism, and result in a substantial increase in LDLC and a substantial decline in HDLC (Silverstolpe *et al.*, 1979). These progestogenic effects are dose-dependent and, at the commonly prescribed approximately equivalent doses (King & Whitehead, 1986), it appears that they negate, or even reverse, the beneficial effects of ERT (Henderson *et al.*, 1988). The addition of a progestogen is therefore likely to be detrimental to the heart disease situation. This does not mean that an LHRHA + EPRT regimen will necessarily increase a woman's risk of heart disease relative to her risk if she used no hormonal regimen, only that her heart disease risk will be higher with LHRHA + EPRT than with LHRHA + ERT.

Another concern about adding a progestogen to ERT is that of breast cancer. Maximum mitotic activity in breast tissue occurs around day 25 of the menstrual cycle at the time of maximum progesterone levels (Ferguson & Anderson, 1981), suggesting that progestogens may be important breast-cell mitogens. In sharp contrast to the marked reduction in endometrial cancer risk with COC use, there is no evidence of a reduced risk of breast cancer even after long periods of COC use. There is, in fact, some evidence that COC use at perimenopausal ages, and possibly at young ages, may even increase breast cancer risk (Henderson *et al.*, 1988). (The reason for this may be that exposure to mitogenic hormones in a perimenopausal woman, and in a frequently anovular young woman, is greater when she is using COCs than when she is not using exogenous hormones.) There is no evidence that adding a progestogen to high-dose LHRHA + ERT will benefit the breast cancer situation, and the mitotic activity data suggest that it may well have a significant deleterious effect.

In the context of post-menopausal ERT, we have argued that if the addition of a progestogen has even a moderate adverse effect on the heart disease component of the risk-benefit equation, then its addition is ill-advised when considered in mortality or 'serious disease' terms (Henderson *et al.*, 1988). Endometrial cancer is a significant clinical concern with ERT use in post-menopausal women and will be so with a high-dose LHRHA + ERT regimen; long-term use of unopposed ERT is therefore likely to be considered clinically unacceptable by some physicians even if the predicted level of risk is comparable to that of a premenopausal woman of similar age using no exogenous hormones. Some 'compromise' is therefore called for in which progestogens are prescribed at the lowest dose for the shortest possible time to achieve the desired histological changes in the endometrium. Twelve days of progestogen therapy appears to be the minimum duration necessary to control endometrial hyperplasia completely (Studd *et al.*, 1980; Whitehead *et al.*, 1983), but there is evidence to suggest that such a regimen is not required every cycle (Schiff *et al.*, 1982); a small proportion of women will develop hyperplasia if progestogens are not given every cycle, but few will develop symptoms, and a 12-day progestogen course every 3–4 cycles will likely eliminate any hyperplasia that has developed (Schiff *et al.*, 1982). Such a regimen will not only benefit the endometrium but will also reduce bone loss. There will be no modification of the beneficial effect on ovarian risk. The cardiovascular situation should remain satisfactory, and any negative effect on breast cancer risk should be sufficiently small as to leave the regimen with a substantial beneficial effect on breast cancer risk.

If alternative progestogen regimens are found (e.g. possibly progestogen patches, or micronised progesterone) (Ottosson *et al.*, 1985; Editorial, 1988; Henderson *et al.*, 1988) that do not adversely affect the beneficial effects of unopposed oestrogen (ERT) on lipoprotein cholesterol, and other possible risk factors (Lin *et al.*, 1982; Makila *et al.*, 1982; Henderson *et al.*, 1988), for cardiovascular disease, then the critical unknown in the comparison of EPRT and ERT will be the effect of progestogen on the risk of breast cancer. Current research on the relation of medroxyprogesterone acetate and low-dose COCs to breast cancer risk will, hopefully, give us some further guidance on this vital issue.

Since the only reason for wanting to add a progestogen to the proposed LHRHA + ERT regimen is to reduce endometrial-cell proliferation, an alternative approach would be to somehow deliver a progestogen solely to the endometrium, thus avoiding any further changes in cardiovascular or breast cancer risk. The 'Progestasert' (Alza Corporation, Palo Alto, CA) intra-uterine device does just this; this progesterone-releasing device suppresses the proliferative activity of the endometrium without apparently affecting serum hormone levels (Hagenfeldt *et al.*, 1977). Whether a regimen of LHRHA + ERT plus a possibly modified Progestasert-type device could be made clinically acceptable and acceptable to women will need to be carefully evaluated: the benefits of such a regimen would be a significant reduction in the incidence of breast, ovarian *and* endometrial cancer.

In the first instance, after the essential evaluation of effects on lipid and bone metabolism and clinical acceptability are carried out, the proposed high-dose LHRHA plus low-dose ERT regimen might be offered to women at particularly high risk of breast cancer, and possibly only after they have completed their child-bearing. Although the beneficial effects of this regimen in the perimenopausal years are likely to be less than in the strictly premenopausal period, 15 years of use starting at age 35 (assuming menopause at 50) is predicted to reduce lifetime breast cancer risk to 49% (compared to 28% in row (iv) of Table I), and if use started at age 30 to 31%.

### Appendix

*Mathematical model of breast cancer*

The calculations shown in Table I are based on the 'model' of breast cancer incidence of Pike *et al.* (1983). In brief, the incidence of breast cancer at age $T$, $I(T)$, can be written

$$I(T) = a[M(T)]^{4.5}$$

where $M(T)$ is proportional to the sum of the average 'effective mitotic rates', $m(t)$, of breast cells from birth to age $T$, i.e. $t$ taking all values from birth to age $T$. This model provides an excellent quantitative description of the age-incidence curve and of the major known risk factors for breast cancer, if $m(t)$ is defined as follows:

$m(t) = 0$ from birth to menarche;
$= 1$ from menarche (taken as occurring at age 13) to first full-term pregnancy (FFTP, taken as occurring at age 22); 2.2 is added to $m(t)$ for the year in which FFTP occurs;
$= 0.7$ from FFTP to age 40;
$= 0.105$ after menopause (last menstrual period, taken as occurring at age 50); and $m(t)$ declines linearly from 40 to menopause.

For the 'average' woman not using ERT, we have

$$M(50) = 9 + 2.2 + 18 \times 0.7 + 10 \times (0.7 + 0.105)/2$$

$$= 27.825$$

and

$$M(70) = 27.825 + 20 \times 0.105$$

$$= 29.925.$$

The calculations for row (i) in Table I were made by assuming that the average effective mitotic rate is reduced from the premenopausal value of 0.7 to the post-menopausal value of 0.105 during the time the high-dose LHRHA regimen (with no ERT) is used.

In the framework of this model, the assumed relative risk of 1.75 for 20 years of ERT use (considered to start at menopause at age 50 and be continuous to age 70 when risk is assessed) implies that

$$M(70;\ \text{ERT for 20 years}) = M^*(70) = 33.888$$

(calculated so that $(M^*(70)/29.925)^{4.5} = 1.75$). But

$$M^*(70) = M(50) + 20 \times m^*$$

where $m^*$ is proportional to the average effective mitotic rate of breast cells on the ERT regimen. Thus

$$m^* = (33.888 - 27.825)/20$$
$$= 0.303$$

The calculations for row (ii) in Table I were made by assuming that the average effective mitotic rate is reduced from 0.7 to 0.303 during the time the high-dose LHRHA + ERT regimen is used.

If such ERT is given for 21 days rather than the assumed 26 days per 28-day cycle, the associated effective mitotic rate is predicted to change from 0.303 to 0.265. This is calculated as follows:

$$0.303 = (26/28) \times m^+ + (2/28) \times 0.105$$

where $m^+$ is the mitotic rate on those days ERT is taken, 0.105 is the normal post-menopausal rate and the ERT is taken for 26 days per 28-day cycle. From this equation, $m^+ = 0.318$; and

$$0.265 = (21/28) \times m^+ + (7/28) \times 0.105$$

The calculations for row (iii) in Table I were made by assuming that the average effective mitotic rate is reduced from 0.7 to 0.265 during the time the high-dose LHRHA + 21-day ERT regimen is used.

If ERT is given at a dose of 0.625 mg CEE, 'low-dose' ERT, for 26 days per 28-day cycle, the associated average effective mitotic rate is predicted to change from 0.303 to 0.237. This is calculated as follows:

$$m^{+} = 0.318 = 0.213 + 0.105$$

0.625 mg CEE is two-thirds the dose of CEE associated with $m^{+}$, thus the effective mitotic rate, $m^{-}$, while actually on low-dose ERT, if proportional to ERT dose is

$$m^{-} = (2/3) \times 0.213 + 0.105$$

$$= 0.247$$

and the average effective mitotic rate over a 28-day cycle is

$$0.237 = (26/28) \times 0.247 + (2/28) \times 0.105.$$

The calculations for row (iv) in Table I were made by assuming that the average effective mitotic rate is reduced from 0.7 to 0.237 during the time the high-dose LHRHA + low-dose ERT regimen is used.

If such low-dose ERT is given for 21 days rather than the assumed 26 days per 28-day cycle, the associated average effective mitotic rate is predicted to change to

$$0.212 = (21/28) \times m^{-} + (7/28) \times 0.105$$

where $m^{-}$ is the effective mitotic rate on those days that low-dose ERT is taken, 0.105 is the normal post-menopausal rate and the ERT is taken for 21 days per 28-day cycle. The calculations for row (v) in Table I were made by assuming that the average effective mitotic rate is reduced from 0.7 to 0.212 during the time the high-dose LHRHA + 21-day low-dose ERT regimen is used.

The relative risks shown in Table I apply specifically to age 70: since the normal increase in $M$ after age 50 is small these relative risks essentially predict the situation for all ages over 50; the predicted relative risks under age 50 are less (protection greater) than the figures shown in Table I.

*Mathematical model of ovarian cancer*

The calculations shown in Table II are based on the 'model' of ovarian cancer incidence of Pike (1987). In brief, the incidence of ovarian cancer at age $T$, $I(T)$, can be written

$$I(T) = a[M(T)]^{4}$$

where $M(T)$ is proportional to the sum of the average 'effective mitotic rates', $m(t)$, of ovarian epithelial cells from birth to age $T$, i.e. $t$ taking all values from birth to age $T$. This model provides an excellent quantitative description of the age-incidence curve and of the major known risk factors for ovarian cancer, if $m(t)$ is defined as follows:

$m(t) = 0.09$ before menarche (taken as occurring at age 13); after menopause (taken as occurring at age 50); and for the periods of anovulation associated with pregnancy and taking COCs;

$= 1$ otherwise.

For an 'average' woman with three children we have

$$M(50) = 50 - 13 - 3 + 0.09 \times (13 + 3)$$

$$= 35.44$$

and

$$M(70) = 35.44 + 20 \times 0.09$$

$$= 37.24.$$

In the framework of this model,

$$M(70; \text{LHRHA for } D \text{ years}) = 37.24 - D + 0.09 \times D$$

$$= 37.24 - 0.91 \times D.$$

The calculations in Table II were made on this basis.

The relative risks shown in Table II apply specifically to age 70; since the normal increase in $M$ after age 50 is small these relative risks essentially predict the situation for all ages over 50; the predicted relative risks under age 50 are less (protection greater) than the figures shown in Table II.

### References

BARNES, R.B., ROY, S. & LOBO, R.A. (1985). Comparison of lipid and androgen levels after conjugated estrogen or depo-medroxyprogesterone acetate treatment in postmenopausal women. *Obstet. Gynecol.*, **66**, 216.

BAIRD, D.D., TYROLER, H.A., HEISS, G., CHAMBLESS, L.E. & HAMES, C.G. (1985). Menopausal change in serum cholesterol. *Am. J. Epidemiol.*, **122**, 982.

BERNSTEIN, L., ROSS, R.K., LOBO, R., HANISCH, R., KRAILO, M.D. & HENDERSON, B.E. (1987). The effects of moderate physical activity on menstrual cycle patterns in adolescence: implications for breast cancer prevention. *Br. J. Cancer*, **55**, 681.

CHRISTIANSEN, C., CHRISTENSEN, M.S. & TRANSBOL, T.B. (1981). Bone mass in postmenopausal women after withdrawal of oestrogen/gestagen replacement therapy. *Lancet*, **i**, 459.

COOK, P.J., DOLL, R. & FELLINGHAM, S.A. (1969). A mathematical model for the age distribution of cancer in man. *Int. J. Cancer*, **4**, 93.

EDITORIAL (1986). Oral contraceptives and breast cancer. *Lancet*, **ii**, 665.

EDITORIAL (1988). Patch up the menopause. *Lancet*, **i**, 861.

ELWOOD, J.M., COLE, P., ROTHMAN, K.J. & KAPLAN, S.D. (1977). Epidemiology of endometrial cancer. *J. Natl Cancer Inst.*, **59**, 1055.

ETTINGER, B., GENANT, H.K. & CANN, C.E. (1987). Postmenopausal bone loss is prevented by treatment with low-dosage estrogen with calcium. *Ann. Intern. Med.*, **106**, 40.

FERGUSON, D.J.P. & ANDERSON, T.J. (1981). Morphological evaluation of cell turnover in relation to the menstrual cycle in the 'resting' human breast. *Br. J. Cancer*, **44**, 177.

FRASER, H.M. & BAIRD, D.T. (1987). Clinical applications of LHRH analogues. *Baillieres Clin. Endocrinol. Metab.*, **1**, 43.

GUDMUNDSSON, J.A., NILLIUS, S.J. & BERGQUIST, C. (1986). Intranasal peptide contraception by inhibition of ovulation with the gonadotropin-releasing hormone superagonist nafarelin: six months' clinical results. *Fertil. Steril.*, **45**, 617.

HAGENFELDT, K., LANDGREN, B.-M., EDSTROM, K. & JOHANNISSON, E. (1977). Biochemical and morphological changes in the human endometrium induced by the progestasert device. *Contraception*, **16**, 183.

HENDERSON, B.E., ROSS, R.K., PIKE, M.C. & CASAGRANDE, J.T. (1982). Endogenous hormones as a major factor in human cancer. *Cancer Res.*, **42**, 3232.

HENDERSON, B.E., ROSS, R.K. & PIKE, M.C. (1983). Exogenous hormones and the risk of cancer. In *Recent Advances in Cancer Control*, Yamagata S., Hirayama, T. & Hiramichi, S. (eds) p. 73. Excerpta Medica: New York.

HENDERSON, B.E., ROSS, R.K., LOBO, R.A., PIKE, M.C. & MACK, T.M. (1988). Re-evaluating the role of progestogen therapy after the menopause. *Fertil. Steril.*, **49**, suppl., 9.

HILDRETH, N.G., KELSEY, J.L., LIVOLSI, V.A. and 5 others (1981). An epidemiological study of epithelial carcinoma of the ovary. *Am. J. Epidemiol.*, **114**, 398.

KANNEL, W.B. & GORDON, T. (1987). Cardiovascular effects of the menopause. In *Menopause: Physiology and Pharmacology*, Mishell, D.R. (ed) p. 91. Year Book: Chicago.

KELSEY, J.L. (1979), A review of the epidemiology of human breast cancer. *Epidemiol. Rev.*, **1**, 74.

KEY, T.J.A. & PIKE, M.C. (1988*a*). The role of oestrogens and progestagens in the epidemiology and prevention of breast cancer. *Eur. J. Cancer Clin. Oncol.* **24**, 29.

KEY, T.J.A. & PIKE, M.C. (1988*b*). The dose-effect relationship between 'unopposed' oestrogens and endometrial mitotic rate: its central role in explaining and predicting endometrial cancer risk. *Br. J. Cancer*, **57**, 205.

KING, R.J.B. & WHITEHEAD, M.I. (1986). Assessment of the potency of orally administered progestins in women. *Fertil. Steril.*, **46**, 1062.

LEWIS, B. (1978). Hormones and lipoprotein metabolism. In *Coronary Heart Disease in Young Women*, Oliver, M.F. (ed) p. 121. Churchill Livingstone: Edinburgh.

LIN, A.L., McGILL, H.C. & SHAIN, S.A. (1982). Hormone receptors of the baboon cardiovascular system. *Circ. Res.*, **50**, 610.

LINDSAY, R., HART, D.M., AITKEN, J.M., MACDONALD, E.B., ANDERSON, J.B. & CLARK, A.C. (1976). Long-term prevention of postmenopausal osteoporosis by oestrogens. *Lancet*, **i**, 1038.

LINDSAY, R., HART, D.M. & CLARK, D.M. (1984). The minimum effective dose of estrogen for prevention of postmenopausal bone loss. *Obstet. Gynecol.*, **63**, 759.

LOBO, R.A. (1987). Prevention of postmenopausal osteoporosis. In *Menopause: Physiology and Pharmacology*, Mishell, D.R. (ed) p. 165. Year Book: Chicago.

McLACHLAN, R.I., HEALY, D.L. & BURGER, H.G. (1986). Clinical aspects of LHRH analogues in gynaecology: a review. *Br. J. Obstet. Gynaecol.*, **93**, 431.

McPHERSON, K. & DRIFE, J.O. (1986). The pill and breast cancer: why the uncertainty? *Br. Med. J.*, **293**, 709.

MAKILA, U.-M., WAHLBERG, L., VIINIKKA, L. & YLIKORKALA, O. (1982). Regulation of prostacyclin and thromboxane production by human umbilical vessels: the effect of estradiol and progesterone in a superfusion model. *Prostaglandins Leukotrienes*, **8**, 115.

MANN, J.I., LEWIS, B., SHEPHERD, J. and 4 others (1988). Blood lipid concentrations and other cardiovascular risk factors: distribution, prevalence, and detection in Britain. *Br. Med. J.*, **296**, 1702.

MOORE, D.H., MOORE, D.H. & MOORE, C.T. (1983). Breast carcinoma etiological factors. *Adv. Cancer Res.*, **40**, 189.

OTTOSSON, U.B., JOHANSSON, B.G. & VON SCHOULTZ, B. (1985). Subfractions of high-density lipoprotein cholesterol during estrogen replacement therapy: a comparison between progestogens and natural progesterone. *Am. J. Obstet. Gynecol.*, **151**, 746.

PAGANINI-HILL, A., ROSS, R.K., GERKINS, V.R., HENDERSON, B.E., ARTHUR, M. & MACK, T.M. (1981). Menopausal estrogen therapy and hip fracture. *Ann. Intern. Med.*, **95**, 28.

PIKE, M.C. (1987). Age-related factors in cancers of the breast, ovary and endometrium. *J. Chron. Dis.*, **40**, suppl. II, 59.

PIKE, M.C., KRAILO, M.D., HENDERSON, B.E., CASAGRANDE, J.T. & HOEL, D.G. (1983). 'Hormonal' risk factors, 'breast tissue age' and the age-incidence of breast cancer. *Nature*, **303**, 767.

ROSENBERG, L., HENNEKENS, C.H., ROSNER, B., BELANGER, C., ROTHMAN, K.J. & SPEIZER, F.E. (1981). Early menopause and the risk of myocardial infarction. *Am. J. Obstet. Gynecol.*, **139**, 47.

SCHIFF, I., SELA, H.K., CRAMER, D., TULCHINSKY, D. & RYAN, K.J. (1982). Endometrial hyperplasia in women on cyclic or continuous estrogen regimens. *Fertil. Steril.*, **37**, 79.

SILVERSTOLPE, G., GUSTAFSON, A., SAMSIOE, G. & SVANBORG, A. (1979). Lipid metabolic studies in oophorectomized women. Effects of three different progestogens. *Acta. Obstet. Gynecol. Scand.*, suppl. **88**, 89.

STADEL, B.V. (1981). Oral contraceptives and cardiovascular disease. *N. Engl. J. Med.*, **305**, 612 & 672.

STUDD, J.W.W., THOM, M.H. & PATERSON, M.E.L. (1980). The prevention and treatment of endometrial pathology in postmenopausal women receiving exogenous oestrogens. In *The Menopause and Postmenopause*, Pasetto, W., Pavletti, R. & Lambrus, J. (eds) p. 127. MTP Press: Lancaster.

THOROGOOD, M., CARTER, R., BENFIELD, L., McPHERSON, K. & MANN, J.I. (1987). Plasma lipids and lipoprotein cholesterol concentrations in people with different diets in Britain. *Br. Med. J.*, **295**, 351.

WAHL, P., WALDEN, C., KNOPP, R. and 4 others (1983). Effects of estrogen/progestin potency on lipid/lipoprotein cholesterol. *N. Engl. J. Med.*, **308**, 862.

WHITEHEAD, M., LANE, G., SIDDLE, N., TOWNSEND, P. & KING, R. (1983). Avoidance of endometrial hyperstimulation in estrogen-treated postmenopausal women. *Semin. Reprod. Endocrinol.*, **1**, 41.